# IL12, IL10, IFNγ and TNFα Expression in Human Primary Monocytes Stimulated with Bacterial Heat Shock GroEL (Hsp64) Protein

**DOI:** 10.1371/journal.pone.0154085

**Published:** 2016-04-27

**Authors:** Ayten Nalbant, Tahsin Saygılı

**Affiliations:** 1 Molecular Immunology and Gene Regulation Laboratory, Department of Molecular Biology and Genetics, Izmir Institute of Technology, Urla, İzmir, 35430 Turkey; 2 Alexion Ilac Ticaret Ltd Sti., Buyukdere Cad. No: 100–102 B Blok Kat:1 Istanbul, 34394 Turkey; University of Iowa Carver College of Medicine, UNITED STATES

## Abstract

*Actinobacillus* (Aggregatibacter) *actinomycetemicomitans* (Aa) is a bacterium that lives in the oral cavity and plays an important role in periodontal diseases. The effect of *A*.*actinomycetemcomitans’s* heat shock family protein GroEL on host or immune cells including monocytes is quite limited. In this study, the recombinant *A*.*actinomycetemcomitans’s* GroEL protein (rAaGroEL) was used as an antigen and its effects on monocytes of peripheral blood mononuclear cells (PBMCs) was investigated. To do that, PBMCs were stimulated with rAaGroEL protein at different time points (16h to 96h) and the cytokines of CD14+ monocytes were detected with intracellular cytokine staining by Flow cytometry. Data showed that AaGroEL protein has an antigenic effect on human primary monocytes. AaGroEL protein responsive CD14 monocytes stimulates the expression of IL12, IL10, IFNγ and TNFα cytokines with different kinetics and expression profile. Therefore, *A*. *actinomycetemcomitans’*s heat shock GroEL protein can modulate innate and adaptive immune responses and contribute to an inflammatory diseases pathology.

## Introduction

Periodontitis is a batch of inflammatory disorders that affect the periodontium. As an important feature of chronic inflammatory peridontal diseases, the bacterial products can activate immune system cells. *A*. *actinomycetemcomitans* which is part of the normal mouth flora is a gram negative bacteria and is strongly associated with human periodontal diseases [1]. *A*. *actinomycetemcomitans* is known to express a number of virulence factors such as Leukotoxin (LTX) and Cytolethal Distending Toxin (CDT) [2,3], which have been both studied extensively. An osteolytic protein of *A*.*actinomycetemcomitans* was identified as a GroEL (Hsp64) protein which belongs to the family of heat shock proteins [4]. The proliferating and cytotoxic activities of *A*.*actinomycetemcomitans’s* GroEL protein on epithelial cells were also reported [5,6]. Recent studies also showed that *Actinobacillus actinomycetemcomitans’s* GroEL protein is a significant inducer of IFNγ and IL10 double cytokine producing T-bet positive T helper type 1 (Th1) cells from CD4 T cells [7].

It is known in the literature that bacterial GroEL proteins from different types of bacteria induces cytokine expression in human host cells and immune system cells [7,8,9,10,11,12,13]. *A*.*actinomycetemcomitans* is among one of the GroEL expressing bacteria. The affect of *A*.*actinomycetemcomitans* GroEL protein on human primary monocytes has not yet been elucidated. Thus, it is important to reveal whether *A*.*actinomycetemcomitans* GroEL protein has a capacity or not to induce human primary monocytes to express cytokines. In this study, we used recombinant *A*.*actinomycetemcomitans* GroEL (rAaGroEL) protein as a model antigen to study human primary monocytes’ immune response. To this extent, human peripheral blood mononuclear cells were cultured with rAaGroEL and cytokine profiles of monocytes were measured. Our data suggested that rAaGroEL stimulated monocytes have a capacity to express different type of cytokines.

## Materials and Methods

### Human Peripheral Blood Mononuclear Cells

Ethics approval for this study was obtained from the Dokuz Eylül University, Noninvasive Research Ethics Committee, İzmir, Turkey. All blood samples were drawn from systemically and periodontal healthy adult donors. A total of 35 voluntary donors were under the age 50 and nonsmokers. All donors were asked to sign an informed consent form which approved by the Noninvasive Ethics Committee of Dokuz Eylül University. Blood samples were drawn from the volunteers at İYTE Health Services by health professionals. Peripheral blood mononuclear cells (PBMC) were isolated by Ficoll-Hypaque density gradient centrifugation [14] from the collected donors’ venous blood.

### Preparation of antigen and stimulation of peripheral mononuclear cells

Previously cloned, expressed and purified recombinant AaGroEL protein was used as an antigen in this study [7]. PBMC cultures had a concentration of 2x10^6^ cells/ml. U bottom cell culture tubes were used for this process. The PBMC cultures were carried out at various time points (16h to 96h). The cultures were incubated at 37°C and with 5% CO_2_. The negative control cells had only RPMI medium. The medium contained RPMI-1640 supplemented with 10% FBS (Bibco), 100 pg/ml streptomycin (Biochrom AG) and 25 mM HEPES buffer (Gibco). PMA (25 ng/ml) concurrently with Ionomycin (1 mg/ml) was used as positive control. PMA mimics DAG which is a PKC activator. Calcium ionophore Ionomycin enhances Ca2+ influx via a store-dependent mechanism. Ionomycin synergizes with PMA in enhancing the activation of PKC. 20 μg/ml of purifed rAaGroEL protein was used as antigen [7]. This is the optimal dose which is needed to activate PBMC. The end of the indicated culture time points, labeling was carried out immediately. Each sample was prepared in triplicates for statistical analysis.

### Intracellular cytokine detection

The PBMC cultures were carried out at various time points (16h to 96h). To detect intracellular cytokines, GolgistopTM (1:1500 dilution, BD Biosciences) was added to the cultures 4h prior to the indicated time points. At the end of the culture time, cells were fixed and permeabilized after CD14 cell surface staining was performed to label monocytes. Then, cytokine antibodies including IL-10, TNFα, IL12 and IFNγ were added to cells. Finally, cytokine labeled cells were analyzed by Flow cytometry. For each cytokine analysis, cells were gated for CD14 positive monocytes.

### Statistical analysis

The data were analyzed by FACSArray system software and FlowJo softwares. Microsoft Office Excel was used for the further analysis of the flow cytometry data. All the samples were carried out in triplicates and the average was taken at each time point and were compared to the negative controls using student’s t test (a two-tailed student’s t test) and p < 0.05 accepted as statistically significant. Error bars represent the standard deviation and * indicates p < 0.05.

## Results

### AaGroEL triggers IL12 cytokine expression

To test the antigenic properties of recombinant AaGroEL protein, cytokine expression profiles of rAaGroEL-stimulated monocytes were investigated. Following stimulation of cells with 20 μg/ml rAaGroEL protein at different time intervals from 0h to 96h, cultured cells were collected at indicated time points. This dose of antigen was previously determined in our studies to trigger the cytokine expression [7]. The simultaneous expression of IL10, IL12, TNFα and IFNγ cytokines was measured with intracellular cytokine staining as detected by flow cytometry.

**Fig 1:**
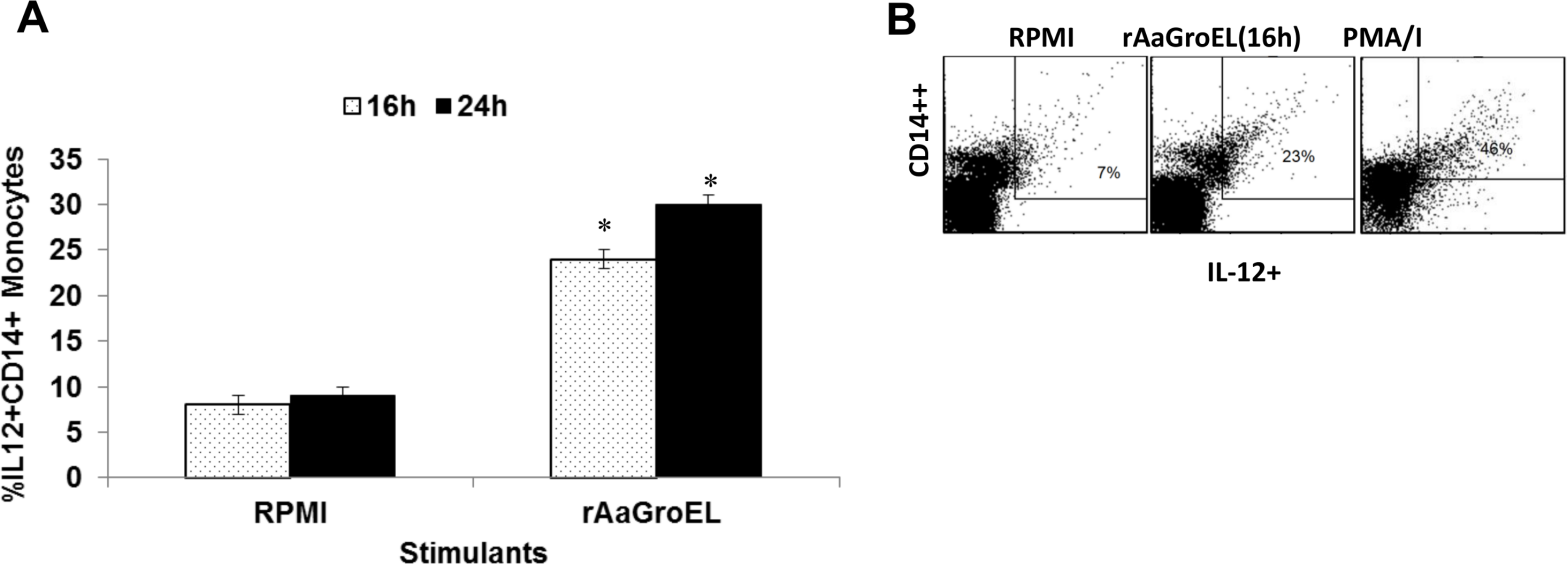
AaGroEL induces IL12 expression. Human PBMCs were cultured with 20μg/ml rAaGroEL protein at 16h and 24h.The RPMI medium and PMA (25 ng/ml) in the presence of Ionomycin (1 mg/ml) were used as negative (NC) and positive controls (PC), respectively. At the indicated time points, CD14 cell surface staining was performed. Then, IL12 intracellular cytokine staining was carried out. Cells were acquired and analyzed. For flow cytometry analysis, cells were gated on monocytes. (A) Expression of IL12 cytokine by CD14+ monocytes. Means and standard deviation (SD) of data were calculated. The data are representative of three independent experiments, where peripheral blood mononuclear cells from a donor were cultured and analyzed in triplicate. Error bars represent the standard deviation and *indicates P<0.05. *(*B) Show representative dot plot analysis of IL12 cytokine expressing monocytes at 16h.

CD14 positive monocytes are antigen presenting cells. Stimulation of monocytes due to antigenic stimulation is crucial to iniate immune responses and to direct naive T cells differentiation. Firstly, IL12 cytokine expression was measured in AaGroEL stimulated monocytes. IL12 is a T cell stimulating factor produced by macrophages, dendritic cells, neutrophils and B cells [15]. It is involved in T cell differentiation to produce Th1 cells. Since IL12 is a polarazing cytokine of CD4 T cells towards Th1, its expresion by AaGroEL responding monocytes was measured at 16h and 24h of cultures. We selected the early time points to measure IL12 expression. Because we previously showed that IFNγ expressing Th1 cells in response to AaGroEL protein reached their highest level at 72h [7]. Data in this study showed that IL12 expressing CD14 monocytes increased up to 23% in AaGroEL-responsive cells compared to 7% in negative controls at 16h. There was a statistically significant 3-fold difference when AaGroEL-treated cells were compared to negative controls at 16h (p < 0.05, Fig 1A and 1B). IL12 cytokine expression was decreased at 24h time point, suggesting a role for IL12 in an early stage of immune response. Fig 1B shows representative dot plot analysis of IL12 expressing monocytes at 16h.

### AaGroEL stimulates IFNγ cytokine expression

**Fig 2:**
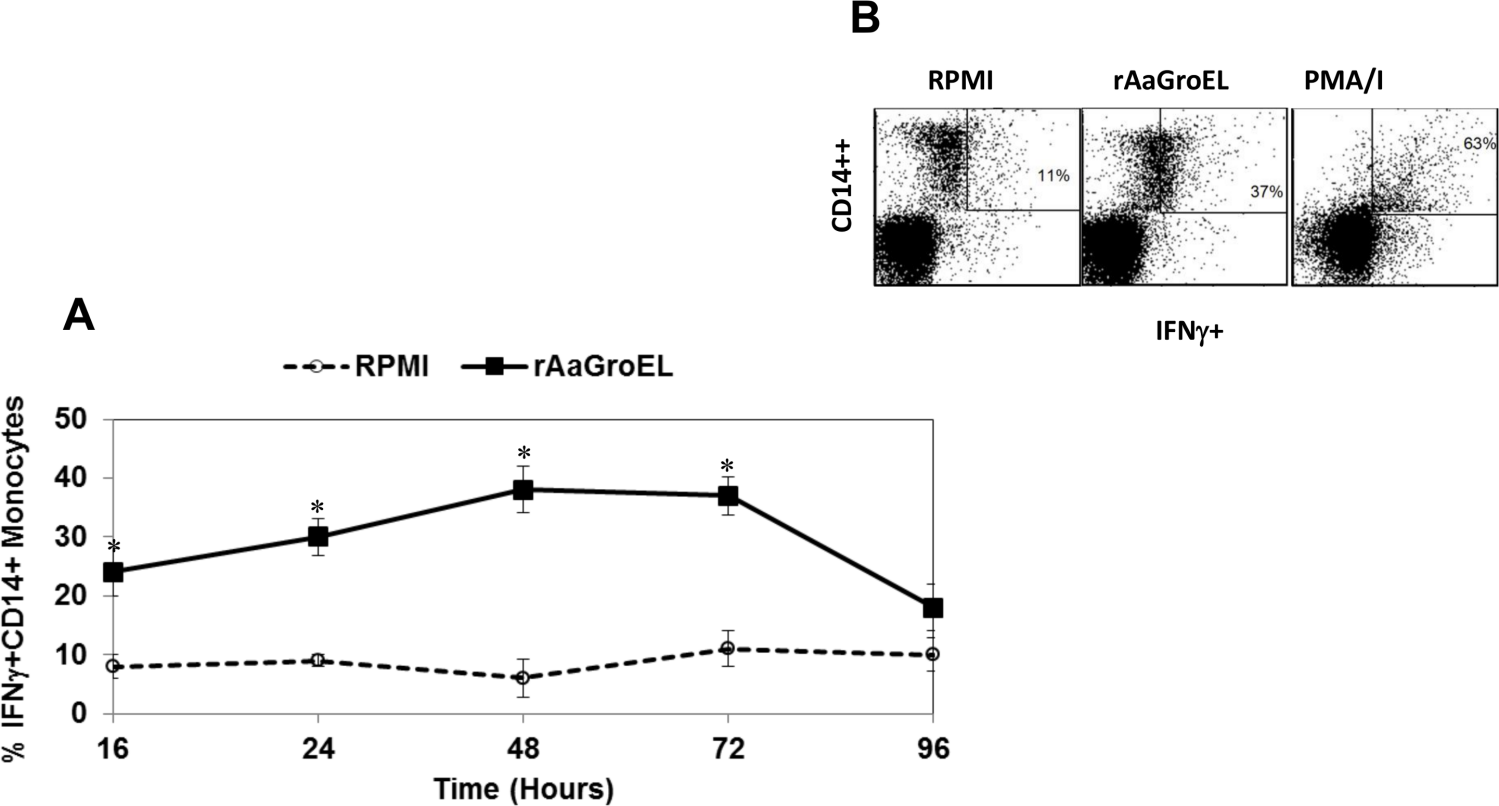
AaGroEL stimulates IFNγ expression. PBMCs were cultured from 16h to 96h with rAaGroEL (20μg/ml). Negative and positive control cultures were carried out as described in material and methods section. At indicated time points, CD14 cell surface staining was performed. Then, IFNγ cytokine antibody was added. Cells were acquired and analyzed. For analysis, cells were gated on CD14 monocytes. (A) Expression of IFNγ by CD14+ monocytes. Means and SD of data were calculated. The data are representative of five independent experiments, where peripheral blood mononuclear cells from a donor were cultured and analyzed in triplicate. Error bars represent the standard deviation and *indicates P<0.05. (B) Show representative dot plot analysis of IFNγ expressing monocytes at 72h.

IFNγ is a pro- and anti-inflammatory cytokine which is regulated by IL12 and IL8. It is produced when antigen specific immune response develops by natural killer (NK) and natural killer T (NKT) cells, CD4 Th1 effector T cells and CD8 T lymphocytes (CTL) [16]. IFNγ is known to shift monocyte differentiation from dendritic cells to macrophages [17]. Next, IFNγ cytokine was measured in rAaGroEL-stimulated cultures. The kinetics of IFNγ cytokine expression data showed that IFNγ was produced at 16h (24%) and slighty increased at 24h (30%). At 48h IFNγ expression reached the highest level (38%). There was a 6-fold increase relative to the negative controls (p< 0.05, Fig 2A). At 96h, the percentage of IFNγ decreased due to the loss of monocytes. The representative dot plot analysis of IFNγ producing monocytes at 72h showed in Fig 2B.

### AaGroEL stimulates IL10 cytokine expression

**Fig 3:**
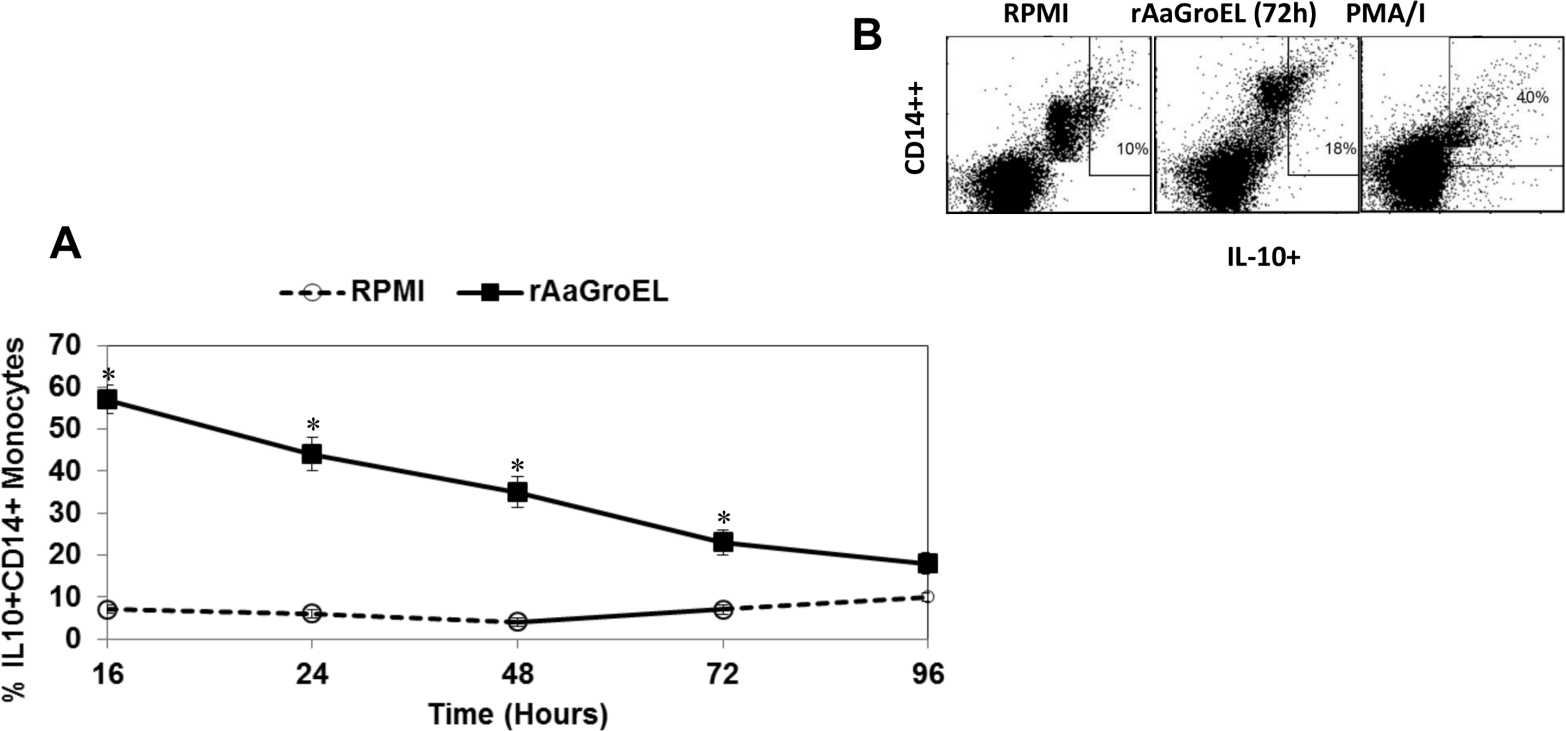
AaGroEL triggers IL10 expression. PBMCs were cultured from 16h to 96h with rAaGroEL (20μg/ml). Negative and positive control cultures were carried out as described in material and methods section. At indicated time points, CD14 cell surface staining was performed. Then, IL10 antibody was added. Cells were acquired and analyzed. For analysis, cells were gated on CD14 monocytes. (A) Expression of IL10 by CD14+ monocytes. Means and SD of data were calculated. The data are representative of five independent experiments, where peripheral blood mononuclear cells from a donor were cultured and analyzed in triplicate. Error bars represent the standard deviation and * P<0.05. (B) Show representative dot plot analysis of IL10 expressing monocytes at 72h.

We next investigated whether rAaGroEL responding monocytes expressed IL10 cytokine. IL10 is a prototypic anti-inflammatory cytokine which is primary produced by macrophages. IL10 has a role in immuneregulation and inflammation. It is known to inhibit IL1, IL6, IL8 and TNFα synthesis in monocytes [18]. Fig 3A shows the percentage of IL10 expressing monocytes in response to AaGroEL at different time points. IL10 had its highest expression at 16h and decreased continuesly at the of 96h. The production of IL10 cytokine reached up to 57% in AaGroEL stimulated cells compared to 7% (p < 0.05, 8-fold increase) in negative control cultures (Fig 3A). The represantative flow cytometry dot plot analysis of IL10 expressing monocytes in response AaGroEL protein as well as negative and positive controls showed in Fig 3B.

### AaGroEL stimulates monocytes to produce TNFα. cytokine

**Fig 4:**
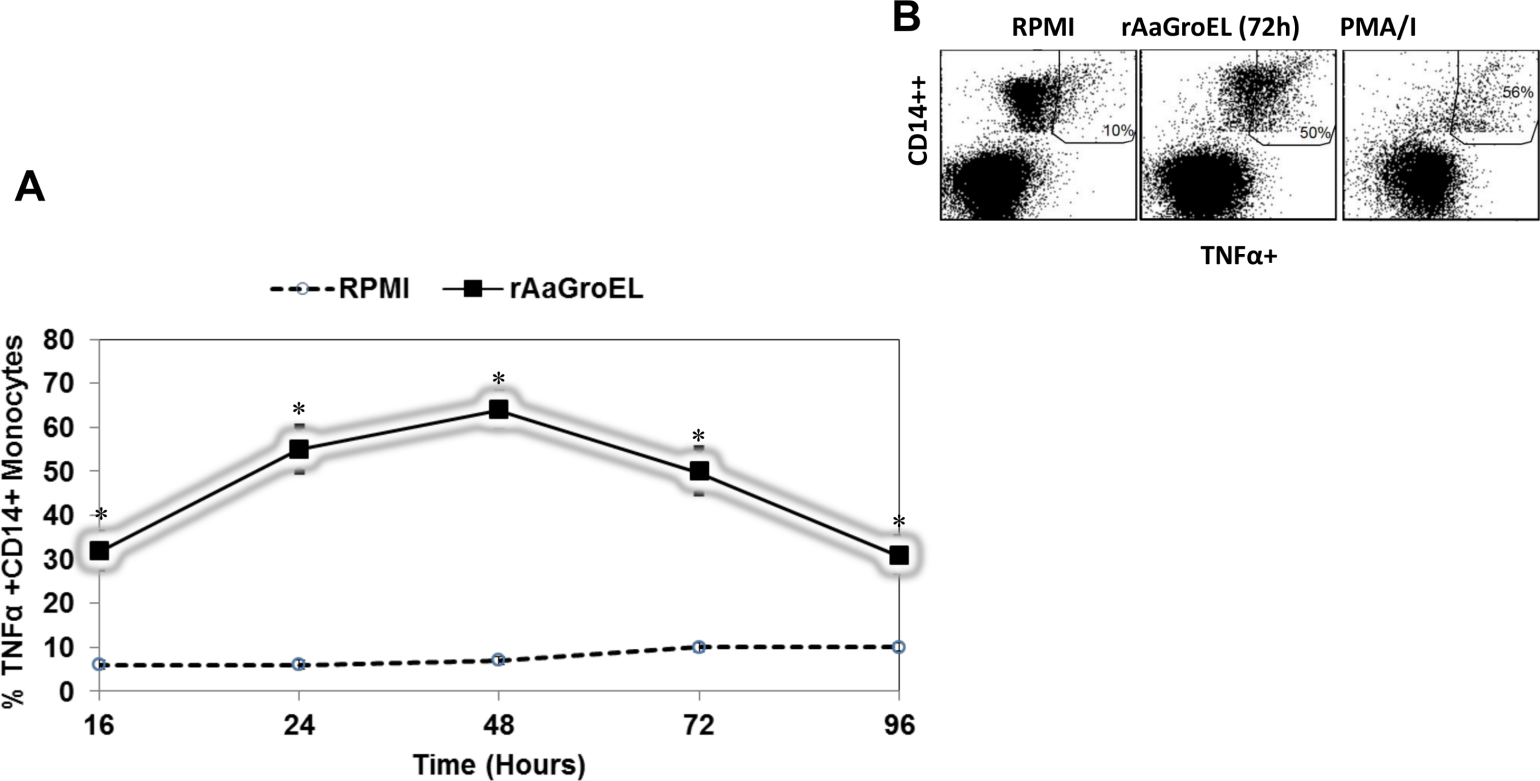
AaGroEL mediates TNFα expression. PBMCs were cultured from 16h to 96h with rAaGroEL (20μg/ml). Negative and positive control cultures were carried out as described in material and methods section. At indicated time points, CD14 cell surface staining was performed. Then, TNFα antibody was added. Cells were acquired and analyzed. For analysis, cells were gated on monocytes. (A) Expression of TNFα by CD14+ monocytes. Means and SD of data were calculated. The data are representative of five independent experiments, where peripheral blood mononuclear cells from a donor were cultured and analyzed in triplicate. Error bars represent the standard deviation and *indicates P<0.05. (B) Show representative dot plot analysis of TNFα expressing monocytes at 72h.

TNFα is produced by macrophages, CD4 lymphocytes, NK cells, neutrophils, eosinophils and mast cells. They are released in response to LPS, IL1 and bacterial products [19]. Therefore, we also investigated the TNFα expression of monocytes in response to AaGroEL protein. Data showed that TNFα production in AaGroEL stimulated monocytes continuesly increased and peaked at 48h. Thereafter, there was a slight decrease up to 96h. The percentages of TNFα expressing AaGroEL stimulated cells were still around 30% at 96 h. The highest level of TNFα expression at 48h was 64%. There was a 9-fold increase relative to the negative controls (7%) (p < 0.05, Fig 4A) at 48h. The representative dot plot analysis of TNFα producing monocytes at 72h showed in Fig 4B.

## Discussion

Heat shock proteins (Hsps) play a fundamental role in folding or unfolding proteins or the maintenance of protein integrity under normal and stressful conditions. Hsp expression is upregulated during stress conditions including infections. It has been also indicated that Hsps might be involved in inflammatory diseases due to their immunogenicity such as chronic inflammatory periodontal diseases [20]. *A*. *actinomycetemcomitans*’s heat shock protein GroEL (Hsp64) is a typical member of bacterial heat shock 60 protein family (Hsp60) and is expressed by this bacterium. The studies in the literature for *A*. *Actinomycetemcomitans*’s GroEL effect on human host [4,5] and immune cells are quite limited [7,20]. We previosly showed that *A*. *actinomycetemcomitans*’s GroEL protein can activate T cells and activated T cells polarized into IL10 and IFNγ producing Tbet positive Th1 cells [7]. It would be very valuable to know the modulation of monocyte immune response by GroEL protein of *A*. *actinomycetemcomitans*. In this study, we investigated the ability of *A*. *actinomycetemcomitans* GroEL protein to stimulate immune response in human primary monocytes. The previously cloned, expressed and purified recombinant AaGroEL was used as antigen to stimulate human PBMCs including monocytes [7]. To our best of knowledge, our data demonstrate for the first time that IL12, INFγ, IL10 and TNFα cytokines were expressed by primary human monocytes stimulated with GroEL protein of *A*. *actinomycetemcomitans*.

Firstly, we showed that *A*. *actinomycetemcomitans* GroEL protein responding monocytes expressed variety of cytokines (Figs 1–4). AaGroEL protein stimulates the monocytes to produce a range of cytokines including IL10, IL12, TNFα and IFNγ. For instance, IL12 cytokine was expressed very early in the monocytes due to GroEL stimulation in the cultures (Fig 1A and 1B). Monocytes/macrophages as antigen presenting cells (APCs) play a key role in T cell function and IL12 is produced by APCs in response to antigenic stimulation and IL12 is also required for polarization of naïve CD4 T cells into an effector Th1 phenotype. As we mentioned earlier, GroEL of *A*. *actinomycetemcomitans* stimulates Th1 phenotype conversion of CD4 T cells [7]. Our finding suggests that AaGroEL stimulated monocytes by expressing IL12 cytokine can contribute to CD4 T cell fate determination during T helper differentiation.

There are known bacterial Hsp’s that stimulates cytokine expression in monocytes/macrophages [9]. For example, *Helicobacter pylori* heat shock protein 60 (H pylori-HSP60) induces IL8 expression in monocytic cells [21] and IL6 production in macrophages utilized by Toll Like Receptor 2 and 4 [22]. Furthermore, *Mycobacterium tuberculosis* infected monocytes produces IL26 cytokines which belong to IL10 cytokine family [23]. Our finding suggests that different bacterial Hsp60s might effect human monocytes differently to express different type of cytokines and *A*. *actinomycetemcomitans*’s GroEL protein is one of them.

We also showed that there is different expression kinetics of each cytokine by CD14 monocytes in response to AaGroEL protein (Figs 1–4). Each of these cytokines can function as pro-inflammatory or anti-inflammatory cytokines. For instance, IFNγ is a pro- and anti-inflammatory cytokine which is regulated by IL12 and IL8. The IFNγ cytokine was expressed at early time points by AaGroEL responding monocytes and slighty increased up to 48h (Fig 2A and 2B). IFNγ can activate macrophages. Moreover, TNFα production in AaGroEL stimulated monocytes continuesly increased and peaked at 48h. There was continuous presence of TNFα up to 96h with a slight decrease (Fig 4A and 4B). Both of the IFNγ and TNFα cytokines presence can increase expression of Class I MHC molecules on many cell types. On the other hand, IL10 is a prototypic anti-inflammatory cytokine which is primarily produced by macrophages. IL10 as a suppressive cytokine can inhibit macrophages activation. It is reasonable to suggest a role for a GroEL protein of *A*. *actinomycetemcomitans* as a chaperone of this bacteria to promote or modulate the inflammatory conditions.

Recombinant GroEL protein of *A*. *actinomycetemcomitans* was previously used in a study to show the role of eukaryotic heat shock protein 60’s (Hsp60) role in inflammatory nature of peridontal diseases. In this study, soluble TNFα expression was measured by ELISA in the monocytic cell line THP1 and was not detected [20]. However, this study did not measure the intracellular cytokine expression with cell surface marker. Additionally, the source of cells and culture settings were quite different. THP1 cells were first stimulated with PMA and then with the antigen of interest. The presented study showed that human primary monocytes stimulated with AaGroEL protein stimulates TNFα cytokine expression through out the cultures (Fig 4A and 4B). Our conclusion is that TNFα expression levels in response AaGroEL protein can differs based on different culture conditions and cells source.

It is also known in the literature that *A*. *actinomycetemcomitans* makes Ltx and CDT and they are considered as virulence factors. There are different ways that bacterial virulence factors interact with host target cells. It was previously shown that the CDT of *A*. *actinomycetemcomitans* blocks PI-3K signaling pathway and induces macrophages to produce proinflammatory cytokines [24]. Moreover, CdtC and CdtB subunits of *A*. *actinomycetemcomitans* CDT interact cholesterol within lipid rafts of plasma membrane [25]. It clearly suggests that different virulence factor of same bacterium like *A*. *actinomycetemcomitans* can be regulated differentially. This allows pathogenic bacteria to utilize different strategies to invade the host.

Taken together, in this study it was shown for first time that *A*. *actinomycetemcomitans* GroEL stimulates IL12, IL10, IFNγ and TNFα cytokines expression in primary human monocytes. Futhermore, production of these cytokines indicates that AaGroEL stimulates monocytes to express pro- and anti-inflammmatory cytokines. Therefore, data suggest that IL12, IFNγ, IL10 and TNFα cytokines produced by monocytes can play a role in immune response modulation and inflammation. In order to further elaborate the importance of monocytes activation and their cytokines in immune response, it is important to determine the mechanisms in which GroEL antigen communicates with monocytes, possibly through utilizing lipid rafts components or the Toll Like Receptors. The immune response modulation capacity of *A*. *actinomycetemcomitans* antigens or virulence factors including GroEL protein can help us to understand the role of *A*.*actinomycetemcomitans* in inflammatory disease conditions.
